# Elevated IL-1β and Comparable IL-1 Receptor Antagonist Levels Are Characteristic Features of L-PRP in Female College Athletes Compared to Male Professional Soccer Players

**DOI:** 10.3390/ijms242417487

**Published:** 2023-12-14

**Authors:** Tomoharu Mochizuki, Takashi Ushiki, Katsuya Suzuki, Misato Sato, Hajime Ishiguro, Tatsuya Suwabe, Satoshi Watanabe, Mutsuaki Edama, Go Omori, Noriaki Yamamoto, Tomoyuki Kawase

**Affiliations:** 1 Department of Orthopaedic Surgery, Graduate School of Medical and Dental Sciences, Niigata University, Niigata 951-8510, Japan; tommochi121710@gmail.com; 2 Division of Hematology and Oncology, Graduate School of Health Sciences, Niigata University, Niigata 951-8518, Japan; tushiki@med.niigata-u.ac.jp; 3 Department of Transfusion Medicine, Cell Therapy and Regenerative Medicine, Niigata University Medical and Dental Hospital, Niigata 951-8520, Japan; katsuyasuzu.xq1@nuh.niigata-u.ac.jp (K.S.); misatosato.zd8@nuh.niigata-u.ac.jp (M.S.); 4 Department of Hematology, Endocrinology and Metabolism, Faculty of Medicine, Niigata University, Niigata 951-8510, Japan; power@med.niigata-u.ac.jp (H.I.); tsuwabe@med.niigata-u.ac.jp (T.S.); 5 Department of Orthopaedic Surgery, Niigata Medical Center, Niigata 950-2022, Japan; wwsatoshiww@gmail.com; 6 Department of Health and Sports, Faculty of Health Sciences, Niigata University of Health and Welfare, Niigata 950-3102, Japan; edama@nuhw.ac.jp (M.E.); omori@nuhw.ac.jp (G.O.); 7 Department of Orthopaedic Surgery, Niigata Rehabilitation Hospital, Niigata 950-3304, Japan; nirehp.yamamoto@aiko.or.jp; 8 Division of Oral Bioengineering, Graduate School of Medical and Dental Sciences, Niigata University, Niigata 951-8514, Japan

**Keywords:** platelet-rich plasma, athletes, interleukin-1β, interleukin-1 receptor antagonist, growth factors

## Abstract

Autologous platelet-rich plasma (PRP) therapy has been becoming popular for the treatment of musculotendinous injuries among athletes. However, for individual and practical variations, clinical success is hardly predictable. To overcome this difficulty, we have been exploring possible criterion candidates for monitoring its clinical effectiveness. In this study, we focused on sex-based differences in young elite athletes and compared the biochemical compositions of their PRP. Leukocyte-rich PRP (L-PRP) was manually prepared from blood samples collected from male professional soccer players (mPSPs) (n = 25) and female college athletes (fCAs) (n = 36). Platelet-derived growth factor-BB (PDGF-BB), transforming-growth factor-β1 (TGFβ1), platelet factor-4 (PF4), interleukin-1β (IL-1β), and IL-1 receptor antagonist (IL-1RA) were quantified using an enzyme-linked immunosorbent assay. The levels of PDGF-BB, TGFβ1, and PF4 in L-PRP were significantly higher in mPSPs than in fCAs. Conversely, IL-1β and IL-1RA were detected at significantly and slightly higher levels, respectively, in fCAs than in mPSPs. Our findings suggest that, even though L-PRP from fCAs may have lower potential to induce cell growth and differentiation than that of mPSPs, due to the latter’s higher capacity to control inflammation, it does not necessarily imply that PRP treatment in fCAs is less effective. Thus, these cytokine levels should be checked before PRP therapy.

## 1. Introduction

Platelet-rich plasma (PRP) is a mixture of concentrated growth factors stored within platelets that stimulate tissue regeneration when injected at the site of injury [1]. PRP therapy has gained widespread acceptance across various fields of regenerative therapy, owing to its inherent biocompatibility, safety, and lack of transmissible disease risks [2,3]. The rapid adoption and application of PRP therapy can also be attributed to its simple and convenient preparation protocols and favorable cost-effectiveness ratio. However, ironically, such rapid dissemination of PRP therapy without rigorous preclinical examinations has distorted the appropriate and in-depth understanding of PRP and produced the controversy of its clinical reliability. It has generally been accepted that the clinical outcome of PRP therapy is mainly due to growth factors concentrated in PRP preparations [4]. In PRP, platelet-derived growth factor, transforming growth factor-β, insulin-like growth factor-I, vascular endothelial growth factor (VEGF), and epidermal growth factor are considered representative growth factors in PRP [1]. However, soluble bioactive factors are not limited to the growth factors in PRP. Angiogenic and pro-inflammatory factors, such as VEGF, interleukin-1 (IL-1), interleukin-6, and tumor necrosis factor-α, are thought to play fundamental roles in neovascularization and inflammation. Other factors, such as serotonin, histamine, dopamine, calcium, and adenosine, may influence the process of wound healing. Furthermore, PRP could contain many more identified and unidentified bioactive factors affecting its effectiveness and pharmacological risk factors. Therefore, we believe that PRP action could be modulated complicatedly by interactions, such as synergistic and antagonistic actions, among those bioactive factors. In addition, these contents depend on the individual and vary with preparation protocols, indicating many more variations in their clinical effectiveness.

Experiences of successful PRP therapy in ordinary adults motivated its induction into the field of sports medicine. Recently, the number of individuals, from children to older individuals, who participate in professional or recreational athletics has considerably increased [5]. Consequently, the incidence and severity of sports injuries have increased. PRP therapy is a conventional treatment modality that has gained popularity for treating musculoskeletal injuries, especially those affecting muscles and tendons. In this case, PRP therapy is not limited to tissue repair alone; in most cases, pain relief is the primary objective of the procedure [5]. Regardless of the purpose, the potential for a more rapid return to sports activity, in contrast to surgical interventions, has made PRP an economically appealing choice, particularly in professional sports [6]. However, controversies surrounding the effectiveness of PRP therapy on athletes have become increasingly apparent, as they have on non-athletic adults too. Several meta-analyses on PRP therapy for sports-related injuries concluded that PRP did not significantly improve physical function or pain relief, with some studies failing to support the current use of PRP [2,4,7,8]. Notably, the majority of published clinical studies are observational in nature and lack well-designed randomized control trials (RCTs) [9]. The challenges in standardizing or classifying PRP quality, patient physical condition, injection protocols, and rehabilitation protocols further complicate the interpretation of clinical data [3,10,11]. In contrast, a report has suggested that PRP treatment may be effective in facilitating the resumption of sports-related activities in young recreational athletes in a relatively shorter period of time [5]. This difference could be pivotal in addressing the core of this issue. To resolve conflicts and prevent ineffective treatments, high-quality evidence should be obtained from well-designed RCTs.

Compared with the non-athletic group, the elite athlete group seems relatively homologous in terms of well-trained skeletal muscles. Thus, we thought that it may be better to characterize the PRP of athletic individuals rather than that of non-athletes for the optimization of PRP therapy. For example, although sex differences have been vigorously investigated in sports medicine, the influence of sex on the effectiveness of PRP treatment is not yet understood. In non-athletic individuals, few studies have addressed these questions or provided insights into the potential reasons for the observed sex differences [12,13,14]. To the best of our knowledge, however, there are no published data available for sex differences in athletes. Considering the well-known physical characteristics of female athletes, i.e., the female athlete triad [15,16], it can be anticipated that PRP quality can be more seriously influenced in female athletes than in male athletes. In a previous study [17], we reported that the bioactive factors involved in controlling inflammation are maintained at higher levels in female athletes.

In this study, we aimed to expand this finding by comparison with male athletes. Simultaneously, we had another purpose, which is more fundamental to our project, which was to propose and establish the diagnostic criteria we believe is necessary to enable successful large-scale RCTs in the near future. Based on the findings obtained from a series of studies [17,18,19,20], we propose several candidate criteria. We believe that persistent efforts will be the only way to establish standardized criteria that can facilitate the precise evaluation of individual PRP quality and the physical conditions of recipients [17]. The standardization of the criteria for PRP quality will contribute to strong, more reliable evidence for PRP therapy in clinical studies and more predictable PRP therapy.

## 2. Results

**Figure 1:**
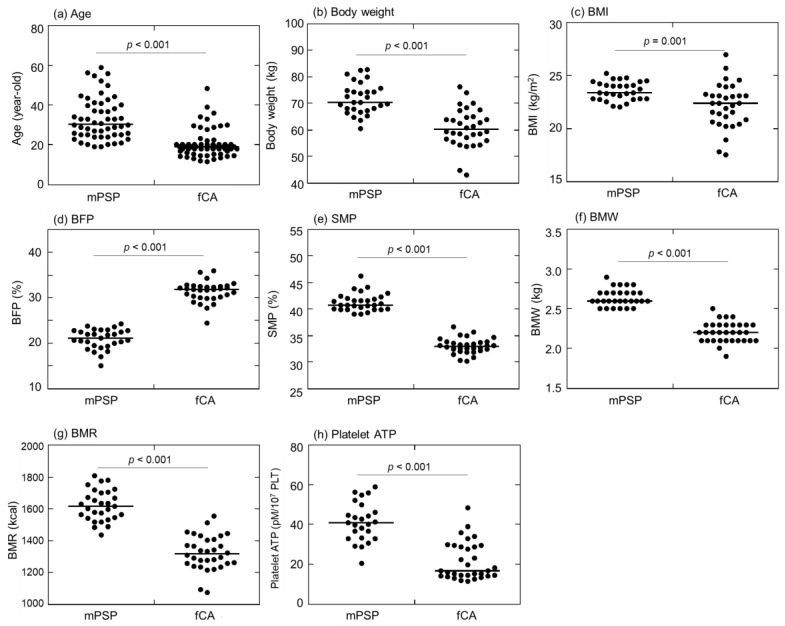
Comparisons of (**a**) age, (**b**) body weight, (**c**) body mass index (BMI), (**d**) body fat percentage (BFP), (**e**) skeletal muscle percentage (SMP), (**f**) bone mass weight (BMW), (**g**) basal metabolic rate (BMR), and (**h**) platelet ATP levels between professional male soccer players (mPSPs) (n = 25) and female college athletes (fCAs) (n = 36). The data of platelet ATP were normalized by platelet (PLT) counts.

Comparisons between mPSPs and fCAs in terms of age, body weight, and BMI are depicted in Figure 1a–c. The median age of male professional soccer players (mPSPs) (25.0 years) was significantly higher than that of fCAs (19.0 years) (Figure 1a). mPSPs had a significantly higher median body weight (70.5 kg) than fCAs (60.2 kg) (Figure 1b). Furthermore, the median value of body mass index (BMI) among mPSPs (23.4 kg/m^2^) was significantly higher than among fCAs (22.4 kg/m^2^) (Figure 1c).

Comparisons of body composition indices (BMIs) and platelet ATP levels between mPSPs and fCAs are depicted in Figure 1d–h. The median value of body fat percentage (BFP) was significantly lower in mPSPs (21.2%) than in fCAs (31.9%) (Figure 1d). The median value of skeletal muscle percentage (SMP) was significantly lower in mPSPs (40.8%) than in fCAs (33.0%) (Figure 1e). The median bone mass weight (BMW) was significantly lower in mPSPs (2.60 kg) than in fCAs (2.20 kg) (Figure 1f). Additionally, the median value of basal metabolic rate (BMR) was significantly lower in mPSPs (1613.0 kcal) than in fCAs (1316.0 kcal) (Figure 1g). Similar data were obtained for platelet ATP levels, with its median value being significantly lower in mPSPs (40.9 pM/10^7^ PLT) than in fCAs (16.9 pM/10^7^ PLT) (Figure 1h).

**Figure 2:**
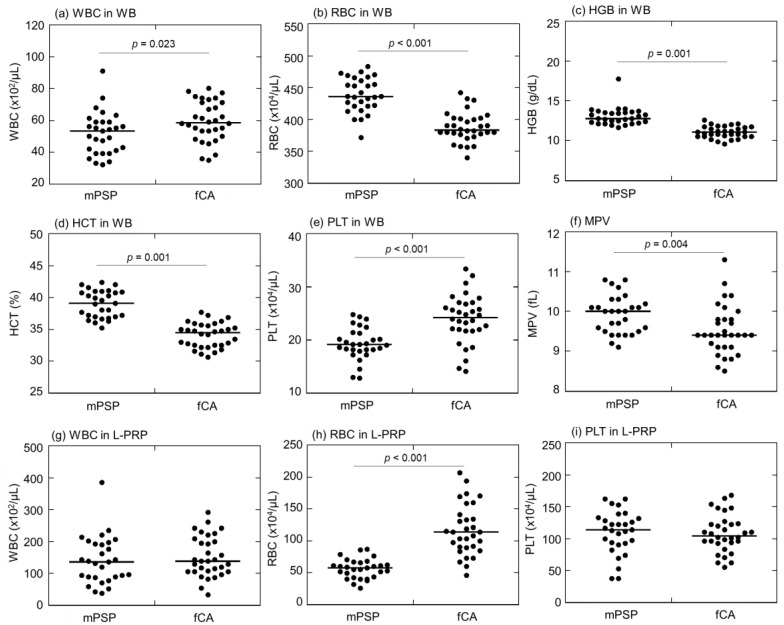
Comparisons of (**a**) white blood cell (WBC) counts, (**b**) red blood cell (RBC) counts, (**c**) hemoglobin (HGB), (**d**) hematocrit (HCT), (**e**) platelet (PLT) counts, and (**f**) mean platelet volume (MPV) in whole blood (WB) samples, and (**g**) WBC counts, (**h**) RBC counts, and (**i**) PLT counts in L-PRP preparations between professional male soccer players (mPSPs) (n = 25) and female college athletes (fCAs) (n = 36).

Comparisons of blood cell counts, red blood cell (RBC)-related parameters, and mean platelet volume (MPV) in whole blood (WB) samples between mPSPs and fCAs are depicted in Figure 2a–f. mPSPs exhibited relatively lower white blood cell (WBC) and platelet counts than those of fCAs (WBC: 53.0 × 10^2^/μL and 58.5 × 10^2^/μL, respectively; PLT: 19.1 × 10^4^/μL and 24.3 × 10^4^/μL, respectively) (Figure 2a,e). For the rest of the parameters, mPSPs exhibited higher values than those of fCAs. The median value of RBC counts was significantly higher in mPSPs (436.0 × 10^4^/μL) than in fCAs (383.0 × 10^4^/μL) (Figure 2b). The medial value of hemoglobin (HGB) was significantly higher in mPSPs (12.8 g/dL) than in fCAs (11.1 g/dL) (Figure 2c). The median value of hematocrit (HCT) was significantly higher in mPSPs (39.1%) than in fCAs (34.5%) (Figure 2d). Furthermore, the median value of MPV was significantly higher in mPSPs (10.0 fL) than in fCAs (9.40 fL) (Figure 2f).

Comparisons of blood cell count in L-PRP preparations between mPSPs and fCAs are depicted in Figure 2g–i. mPSPs (135.0 × 10^4^/μL) had significantly lower median RBC counts than those of fCAs (138.0 × 10^4^/μL) (Figure 2h), while significant differences were observed in both WBC and platelet counts (WBC: 135.0 vs. 138.0 × 10^2^/μL; platelet: 113.9 vs. 104.8 × 10^4^/μL) (Figure 2g,i).

The same L-PRP preparation protocol was applied to both groups. Although PLTs were well concentrated, the concentration rates in RBCs and PLTs were significantly different (Table 1).

**Figure 3:**
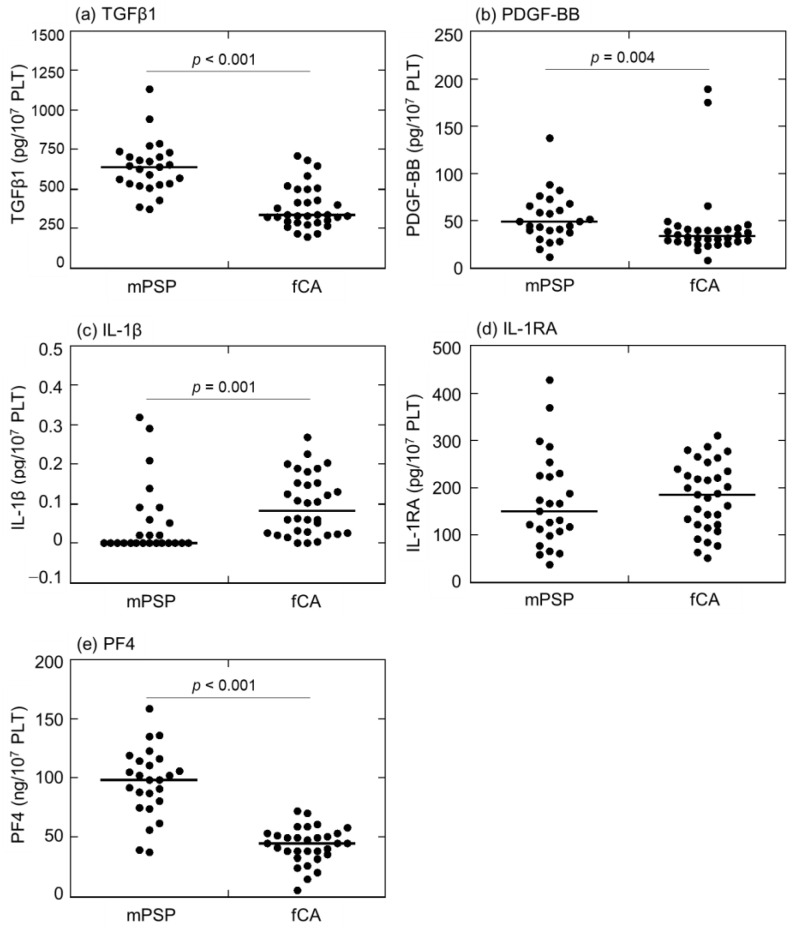
Comparisons of (**a**) transforming-growth factor-β1 (TGFβ1), (**b**) platelet-derived growth factor-BB (PDGF-BB), (**c**) interleukin-1β (IL-1β), (**d**) interleukin-1 receptor antagonist (IL-1RA), and (**e**) platelet factor-4 (PF4) levels (per platelet [PLT] count) in leukocyte- and platelet-rich plasma (L-PRP) preparations between professional male soccer players (mPSPs) (n = 25) and female college athletes (fCAs) (n = 36). The data were normalized by platelet counts.

Comparisons of the growth factors (TGFβ1, PDGF-BB) and cytokines (IL-1β, IL-1RA, and PF4) levels (per platelet [PLT] count) in L-PRP preparations between mPSPs and fCAs are depicted in Figure 3. mPSPs exhibited relatively lower levels of inflammatory cytokines than those of fCAs, whereas, in the rest of the parameters, this relationship was reversed. The median value of TGFβ1 was higher in mPSPs (636.9 pg/10^7^ PLT) than in fCAs (332.5 pg/10^7^ PLT) (Figure 3a). The median value of PDGF-BB was significantly higher in mPSPs (48.9 pg/10^7^ PLT) than in fCAs (33.4 pg/10^7^ PLT) (Figure 3b). In contrast, the median value of IL-1β was significantly lower in mPSPs (<0.0 pg/10^7^ PLT) than in fCAs (0.082 pg/10^7^ PLT) (Figure 3c). The median value of IL-1RA was slightly lower in mPSPs (149.2 pg/10^7^ PLT) than in fCAs (186.1 pg/10^7^ PLT) (Figure 3d). However, the median value of PF4 level was significantly higher in mPSPs (98.6 ng/10^7^ PLT) than in fCAs (44.6 pg/10^7^ PLT) (Figure 3e).

**Figure 4:**
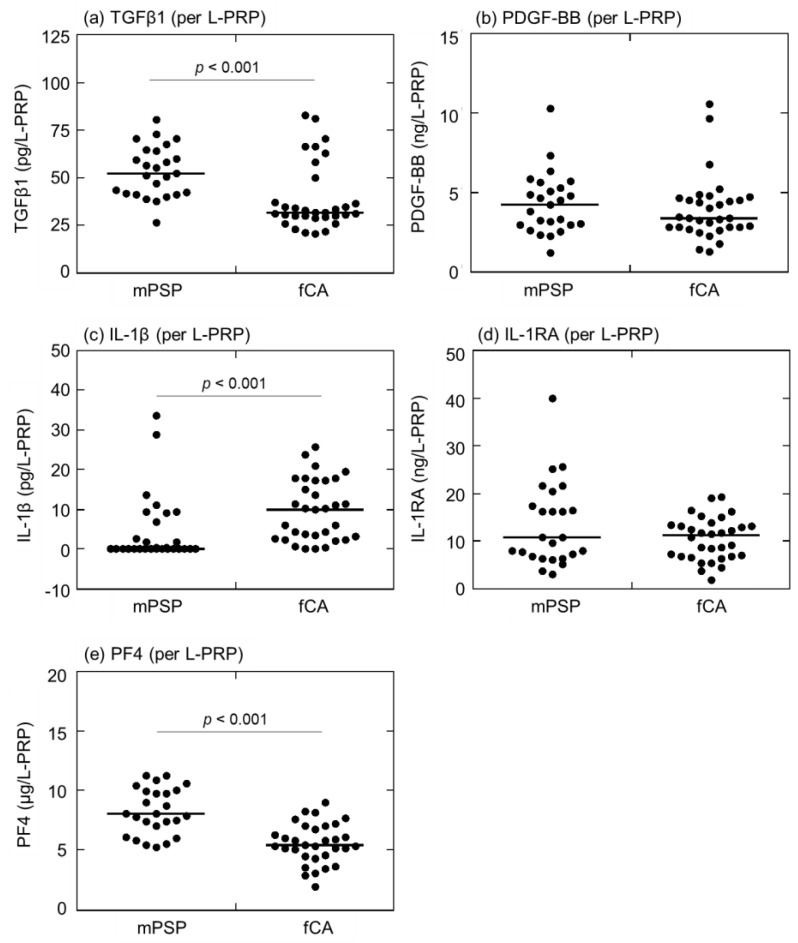
Comparisons of (**a**) transforming-growth factor-β1 (TGFβ1), (**b**) platelet-derived growth factor-BB (PDGF-BB), (**c**) interleukin-1β (IL-1β), (**d**) interleukin-1 receptor antagonist (IL-1RA), and (**e**) platelet factor-4 (PF4) levels (per L-PRP preparation) in leukocyte- and platelet-rich plasma (L-PRP) preparations between professional male soccer players (mPSPs) (n = 25) and female college athletes (fCAs) (n = 36).

Comparisons of growth factors (TGFβ1, PDGF-BB) and cytokines (IL-1β, IL-1RA, and PF4) levels (per L-PRP preparation) in L-PRP preparations between mPSPs and fCAs are depicted in Figure 4. The data obtained show a similar trend. The median value of TGFβ1 was significantly higher in mPSPs (52.1 pg/L-PRP) than in fCAs (31.5 pg/L-PRP) (Figure 4a). The median value of PDGF-BB in mPSPs (4.20 pg/L-PRP) was almost equal to that in fCAs (3.38 pg/L-PRP) (Figure 4b). In addition, the median value of IL-1β was significantly lower in mPSPs (<0.0 pg/L-PRP) than in fCAs (10.1 pg/L-PRP) (Figure 4c). The median IL-1RA levels were almost equal between mPSPs (10.6 ng/L-PRP) and fCAs (11.1 ng/L-PRP) (Figure 4d). Furthermore, the median value of PF4 was significantly higher in mPSPs (8.01 μg/L-PRP) than in fCAs (5.40 μg/L-PRP) (Figure 4e).

The correlation coefficients between BCIs and growth factors or cytokines are summarized in Table 2. mPSPs were characterized by medium correlations of TGFβ1 levels with SMP, BFP, BMR, and BMW. In contrast, fCAs were characterized by weak correlations between IL-1β levels and all the BCIs.

The correlation coefficients between the blood cell counts and growth factors or cytokines are summarized in Table 3. In both groups, IL-1β and IL-1RA levels were strongly or strongly positively correlated with WBC counts. In addition, medium correlations were observed between TGFβ1 levels and PLT counts. The correlations between PF4 or PDGF-BB levels and PLT counts were more pronounced in the fCA group than in the mPSP group.

## 3. Discussion

### 3.1. Definition of “Athlete” in This Study

The term “athlete” is generally well understood and does not require an explicit definition by health professionals [21]. However, for the benefit of medical and scientific investigators, it can be valuable to provide specific criteria or classification to elucidate the concept of “athlete”. For instance, first, information regarding the type of sport or modality (e.g., basketball, football, tennis, swimming, track, and field) and position or characteristics within that sport (e.g., goalkeeper or defender), roughly reflects the quantity and quality of motions required. Second, sex (male and female), age or age categories (young, adult, master, middle-aged, old, college, etc.), nature of participation (recreational and competitive), and type of predominant metabolic pathway for energy production or muscle action (strength and endurance) should be considered the most common criteria to further identify the characteristics and requirements of athletes [21].

In this study, the male group consisted of professional athletes involved in competitive soccer games, actively engaged in regular exercise to increase both strength and endurance and typically below 40 years of age. In contrast, the female group comprised college athletes participating in various sports, within a limited age range. Although their sports experience and technical skills may be inferior to those of mPSPs, fCAs also undergo rigorous training similar to that of mPSPs. Therefore, both groups of athletes shared a common characteristic that could easily be monitored and identified—resting heart rate. It is a well-accepted fact that resting heart rate is significantly reduced by regular exercise [22]. The athletes in both groups exhibited similarly reduced heart rates (lower than 60 bpm) in resting state.

Although the chronological age and sports experiences were different between both groups, we evaluated that these differences were less influential than sex-based differences and “biological” age [23] and determined that these two groups were compatible and appropriate for the primary focus of our study, which aimed to investigate sex-based differences and similarities.

### 3.2. Differences and Similarities between mPSPs and fCAs

In previous studies [17,18,20], regardless of sex, the levels of PRP-representative growth factors were significantly lower in athletes than in age-matched non-athletic adults. This phenomenon can be attributed to the physiological response to exercise, which extends beyond sports. Exercise induces damage to muscle fibers, triggering a spontaneous repair mechanism that consistently involves the mobilization of platelets. Therefore, growth factors and other bioactive molecules stored in platelets can be reduced or exhausted due to repeated, reversible activation.

In this study, mPSPs exhibited higher levels of platelet-associated factors in their L-PRP preparations than those of fCAs, whereas the levels of inflammatory cytokines in fCAs were higher than or comparable to those in mPSPs. The major difference between these findings lies in the source of these bioactive molecules. Specifically, TGFβ1, PDGF-BB, and PF4 are derived from platelets, whereas IL-1β and IL-1RA are derived from WBC [24]. Notably, IL-1β is produced and secreted in response to inflammation but not consistently stored [25]. Secretory IL-1RA is a glycoprotein secreted by activated monocytes and macrophages; two intracellular forms, IL-1RA I and II, are expressed in several types of epithelial cells, fibroblasts, and neutrophils [26]. Therefore, although WBCs are involved in the repair mechanism, these cytokines are rarely exhausted after repeated activation.

The correlation data revealed some notable points: (1) both PF4 and PDGF-BB are preserved at higher levels in fCAs. (2) TGFβ1 levels are correlated with BCIs more substantially in mPSPs. (3) In contrast, IL-1β levels were correlated with BCIs. These findings suggest that mPSPs’ platelets may be repeatedly activated to secrete growth factors in circulation. In addition, inflammatory activity is significantly influenced by BCIs in fCAs.

### 3.3. Lower Levels of Growth Factors in fCAs’ L-PRP

The observed lower levels of growth factor levels in fCAs than in mPSPs raise questions about the underlying reasons for this discrepancy. To interpret these differences effectively, it is essential to evaluate data related to newborn platelets. Platelets have a brief lifespan in circulation, approximately 10 days in humans, and are continuously replenished by bone-marrow-residing megakaryocytes, at a rate of approximately 100 billion platelets per day [27]. Unlike RBCs and WBCs, no sex-based differences in the standard range of platelet counts, MPV, or PDW in peripheral blood have been reported in Japanese populations [28]. Thus, platelets are generally assumed to have a similar lifecycle from generation to cell death in ordinary, non-athletic, and healthy Japanese adults, regardless of sex. However, in this study, fCAs exhibited significantly greater platelet counts than those of mPSPs.

Although other possibilities cannot be ruled out, considering the smaller size (MPV) and lower ATP levels of platelets in fCAs, it is most plausible that the platelet lifespan is prolonged in fCAs, which could result in reduced growth factors stored in platelets after repeated, reversible activation in the circulatory system. When compared with male platelets, due to estrogen, female platelets generally have higher aggregation and activation potential to external stimuli [29,30], indicating that more frequent growth factor release happens in female platelets. As expected, both TGFβ1 and PF4 appeared to be maintained at significantly lower levels in fCAs than in mPSPs. Although a significant difference was not detected, PDGF-PP levels (per L-PRP) tended to be lower in fCAs than in mPSPs.

Because of the difference in baseline blood parameters between males and females [31], PRP composition can be significantly influenced by sex [12,14]. However, there are some conflicting reports regarding sex-based differences in platelet concentrations or growth factor levels [13,32,33], which are often attributed to variations in characteristics of the cohort, preparation protocols, and operators’ skills. Although they are subtle and less influential, these differences along with other minor biases can collectively influence the interpretation of the results. In addition, individual variations in platelet function are known to be more significant than those in other cell types [34,35]. Thus, larger sample sizes may be required to reach a conclusion about sex-based differences to validate our data interpretation.

### 3.4. Higher Levels of Inflammation-Related Cytokines in fCAs’ L-PRP

Studies exploring sex-based differences in proinflammatory and anti-inflammatory cytokine levels in PRP from healthy adults are limited. In this study, we examined only IL-1β and IL-1RA levels (per platelet [PLT] count); thus, further investigations are needed to provide a comprehensive understanding of these sex-based differences. However, the existing literature indicates that female athletes exhibit greater inflammatory responses [36,37,38], which may indicate possible differences in these cytokine levels in PRP, especially leukocyte-containing PRP, L-PRP. If other proinflammatory and anti-inflammatory cytokine levels align with the trends observed for IL-1β and IL-1RA levels, it may suggest that although fCAs have significantly lower levels of representative growth factors in their PRP, tissue regeneration might proceed at a faster, or at least equal, rate than that in mPSPs by effectively controlling the inflammatory phase of the healing process. Notably, although women have a higher capacity to induce inflammatory responses, they also possess a higher capacity to resolve inflammation [39,40].

### 3.5. Clinical Relevance: Factors Influencing Clinical Outcomes of PRP Treatment

The clinical outcomes of PRP treatments are generally believed to be predominantly influenced by the levels of growth factor in PRP. Recent systematic reviews and meta-analyses have raised questions regarding the effectiveness of PRP treatments [2,4,7,8]; however, many clinicians still attribute unsuccessful PRP treatments to variations in patients’ characteristics, operators’ skill, preparation protocols, or treatment protocols. Although these possibilities are indeed important and should be taken into account, other possibilities should also be considered. For instance, cellular responses to external signals, such as growth factors, depend not only on the dose of the signals but also on the cellular capacity as a population. Excess amounts of growth factors beyond the “upper threshold” of full cellular responses may not induce further cellular responses. Furthermore, many cell types undergo receptor internalization, which reduces cellular responses to external growth signals [41]. To overcome these challenges, various drug delivery systems have been developed for regenerative therapies [42].

Therefore, the shortsighted interpretation of the data obtained from comparative studies is unlikely to improve the understanding of PRP and could impede progress. It is more important to analyze the balance of biochemical compositions to evaluate PRP quality than to directly compare the individual compositions. To successfully perform large-sized RCTs in the near future and work toward our ultimate goal, we have been striving to identify possible parameters for a more precise evaluation of PRP quality [17,18,19,20].

## 4. Materials and Methods

### 4.1. Participants and Study Design

A cross-sectional study was conducted involving an independent group of mPSPs (n = 25; age = 19–37 years), who were mainly Japanese and played in the domestic professional soccer league (J1 League), and fCAs (n = 36; age = 18–22 years), who received dietary counseling. The inclusion criteria were as follows: no history of smoking, no systemic diseases regardless of medical control, engagement in daily physical training or games during the regular season, and agreement to provide informed consent. None of the participants were treated with PRP since our study was neither an interventional nor an observational clinical study.

All the participants were evaluated as elite athletes. According to our definition, “elite athletes” have achieved excellent careers in official competitions or something similar. They can be distinguished from those who enjoy recreational sports regardless of their income based on their sports activities.

The study design and consent forms for all procedures (approval no. 2021-0126) were approved by the Ethics Committee for Human Participants of Niigata University, Niigata, Japan. The study was conducted in accordance with the principles outlined in the Declaration of Helsinki, 1964, as revised in 2013. All participants signed informed consent forms.

### 4.2. Blood Collection and Preparation of L-PRP

Blood samples were collected from the participants during non-meal times, specifically avoiding sampling within approximately 1 h of breakfast or lunch. The sample was collected in glass vacuum tubes containing acid-citrate-dextrose (Vacutainer, Becton, Dickinson, and Company, Franklin Lakes, NJ, USA), following previously established procedures [17,20]. The whole blood samples were then transported from the hospitals to the laboratory using a parcel delivery service, maintaining ambient temperatures (approximately 3–10 °C). The pre-warmed blood samples were then processed to prepare L-PRP using the double-spin method, which was performed by well-trained operators. The blood samples were centrifuged at 415× *g* for 10 min, during which the buffy coat was collected at the bottom of the platelet-poor plasma fraction, while separating the upper RBC fraction. A second centrifugation was performed at 664× *g* for 3 min to adjust the volume of L-PRP to approximately 0.9 mL. Both preparations were kept inactive and stored at −80 °C until use [17,20].

### 4.3. Blood Cell Counting

Blood cell counts were performed using an automated hematology analyzer (pocH iV-diff, Sysmex Corporation, Kobe, Japan) [17,20]. In addition to cell counting, data on hematocrit (HCT) and hemoglobin (HGB), mean platelet volume (MPV), and platelet distribution width (PDW) were obtained. Cell counts in whole blood samples were expressed as concentration (cell counts/μL).

### 4.4. Determination of Growth Factor and Cytokine Levels Using Enzyme-Linked Immunosorbent Assay (ELISA)

As previously described [17,18], the concentrations of PDGF-BB, platelet factor 4 (PF4), TGFβ1, IL-1β, and IL-1RA in frozen L-PRP were determined using corresponding human Quantikine ELISA kits (R&D Systems, Inc., Minneapolis, MN, USA). Data obtained for growth factors or cytokines were normalized by both PLT (per PLT: normalized unit PLT counts) or expressed as total amounts in L-PRP preparations (per L-PRP: normalized unit whole L-PRP preparations).

### 4.5. Determination of Platelet ATP Levels

Non-fixed living platelets suspended in Dulbecco’s phosphate buffer saline (PBS) (100 μL) were adjusted to a density ranging between 40 and 60 × 104/µL and stored at −80 °C until needed, typically within 2 weeks. Upon thawing, platelet ATP levels were determined using a luminescence ATP assay kit (FUJIFILM Wako Pure Chemical Corporation, Osaka, Japan) and a luminescencer (AB-2200, Atto Corp., Tokyo, Japan) [20].

### 4.6. Determination of Body Composition

Before blood collection, the body composition of the participants was determined using a bathroom weighing scale (HCS-FS03; ECLEAR, ELECOM, Osaka, Japan). This scale was installed with a unique magnetic resonance imaging-based program that accurately evaluates BFP based on measured bioelectrical impedance and body weight (So et al., 2012 [43]). The BMI, BFP, SMP, BMW, and BMR were automatically determined using this weighing scale.

### 4.7. Statistical Analysis

To compare each parameter between the two groups, data were presented in dot plots with a horizontal bar representing the median value. The presentation was created using a KaleidaGraph (ver 5.01; Synergy Software, Reading, PA, USA). The Mann–Whitney U test was performed to confirm statistical differences in the median and distribution (SigmaPlot version 14.5; Systat Software, Inc., Palo Alto, CA, USA). Differences were considered statistically significant at *p* < 0.05.

Pearson’s correlation analysis was performed to compare the correlation between the two indices, and the correlation coefficients were calculated using SigmaPlot software. The strength of the correlation was defined as very strong (0.8–1.0), strong (0.6–0.79), moderate (0.4–0.59), weak (0.2–0.39), and very weak (0–0.19).

## 5. Conclusions

Our study findings indicated that mPSPs exhibited higher levels of TGFβ1, PDGF-BB, and PF4 in L-PRP preparations than those of fCAs. However, this pattern was not observed for IL-1β and IL-1RA. Therefore, even though L-PRP from fCAs may have a lower potential to induce cell growth and differentiation than that of mPSPs, due to the latter’s higher capacity to control inflammation, it does not necessarily imply that PRP treatment in fCAs is less effective. To enhance the reliability of future RCTs, we recommend including these cytokines as “MUST-CHECK” parameters.

## Figures and Tables

**Table 1:** Comparisons of blood cell concentration rates between professional male soccer players (mPSPs) and female college athletes (fCAs).

Cell Types	mPSP (-Fold)	fCA (-Fold)	*p*
WBC	2.649 ± 1.097	2.547 ± 0.831	0.891
RBC	0.128 ± 0.034	0.314 ± 0.123	<0.001
PLT	5.682 ± 1.470	9.754 ± 2.796	<0.001

The numbers of mPSPs and fCAs were 25 and 36, respectively.

**Table 2:** Correlation coefficients between body composition indices (BCIs) and growth factors or cytokines.

(a) Male Professional Soccer Players (mPSPs)					
mPSP	TGFβ1 (ng/mL)	PDGF-BB (pg/mL)	PF4 (ng/mL)	IL-1β (pg/mL)	IL-1RA (pg/mL)
BMI	−0.160	−0.100	−0.214	−0.214	−0.098
BFP (%)	−0.436	−0.125	−0.187	−0.210	−0.322
SMP (%)	0.429	0.160	0.156	0.179	0.307
BMW (kg)	−0.239	0.107	−0.108	−0.139	−0.167
BMR (kcal)	−0.414	0.030	−0.248	−0.193	−0.249
**(b) Female College Athletes (fCAs)**					
**fCA**	**TGFβ1 (ng/mL)**	**PDGF-BB (pg/mL)**	**PF4 (ng/mL)**	**IL-1β (pg/mL)**	**IL-1RA (pg/mL)**
BMI	0.328	0.229	0.157	0.323	0.175
BFP (%)	0.076	0.007	0.087	0.250	0.066
SMP (%)	−0.090	−0.061	−0.078	−0.219	−0.039
BMW (kg)	0.096	0.057	0.147	0.200	0.185
BMR (kcal)	0.039	−0.004	0.120	0.222	0.148
**(c) The Strength of the Correlation in Both Groups**					
**Strength of Correlation**		**mPSP**		**fCA**	
Very strong (0.8–1.0)		none		none	
Strong (0.6–0.79)		none		none	
Medium (0.4–0.59)		Positive	TGFβ1 vs. SMP	none	
		Negative	TGFβ1 vs. BFP TGFβ1 vs. BMR		
Weak (0.2–0.39)		Positive	TGFβ1 vs. BMW PF4 vs. BMI IL-1RA vs. SMP	Positive	TGFβ1 vs. BMI PDGF-BB vs. BMI IL-1β vs. BMI IL-1β vs. BFP IL-1β vs. BMW IL-1β vs. BMR
		Negative	PF4 vs. BMR IL-1β vs. BMI IL-1β vs. BFP IL-1RA vs. BFP IL-1RA vs. BMR	Negative	IL-1β vs. SMP
Very weak (0–0.19)		others		others	

**Table 3:** Correlation coefficients between blood cell counts and growth factors or cytokines.

(a) Male Professional Soccer Players (mPSPs)					
mPSP	TGFβ1	PDGF-BB	PF4	IL-1β	IL-1RA
WBC (×10^2^/μL)	0.280	−0.094	0.303	0.699	0.934
PLT (×10^4^/μL)	0.480	0.012	0.375	0.509	0.395
**(b) Female College Athletes (fCAs)**					
**fCA**	**TGFβ1**	**PDGF-BB**	**PF4**	**IL-1β**	**IL-1RA**
WBC (×10^2^/μL)	0.388	0.306	0.706	0.788	0.944
PLT (×10^4^/μL)	0.609	0.310	0.649	0.309	0.457
**(c) The Strength of the Correlation in Both Groups**					
**Strength of Correlation**		**mPSP**		**fCA**	
Very strong (0.8–1.0)		Positive	IL-1RA vs. WBC	Positive	IL-1RA vs. WBC
Strong (0.6–0.79)		Positive	IL-1β vs. WBC	Positive	PF4 vs. WBC PF4 vs. PLT IL-1β vs. WBC
Medium (0.4–0.59)		Positive	TGFβ1 vs. PLT IL-1β vs. PLT	Positive	TGFβ1 vs. PLT IL-1RA vs. PLT
Weak (0.2–0.39)		Positive	TGFβ1 vs. WBC PF4 vs. WBC PF4 vs. PLT IL-1RA vs. PLT	Positive	TGFβ1 vs. WBC PDGF-BB vs. WBC PDGF-BB vs. PLT IL-1β vs. PLT
Very weak (0–0.19)		others		none	

## Data Availability

Data are available from the corresponding author upon request.

## Abbreviations

BCI	body composition index
BFP	body fat percentage
BMR	basal metabolic rate
BMW	bone mass weight
fCA	female college athlete
HCT	hematocrit
HGB	hemoglobin
IL-1β	interleukin-1β
IL-1RA	interleukin-1 receptor antagonist
L-PRP	leukocyte- and platelet-rich plasma
mPSP	male professional soccer player
MPV	mean platelet volume
PBS	Dulbecco’s phosphate buffer saline
PDGF-BB	platelet-derived growth factor-BB
PDW	platelet distribution width
PF4	platelet factor 4
PLT	platelet
PRP	platelet-rich plasma
RBC	red blood cell
RCT	randomized controlled trial
SMP	skeletal muscle percentage
TGFβ1	transforming-growth factor-β1
VEGF	vascular endothelial growth factor
WBC	white blood cell
